# Contemporary Advances in Cardiac Remote Monitoring: A Comprehensive, Updated Mini-Review

**DOI:** 10.3390/medicina60050819

**Published:** 2024-05-16

**Authors:** Alberto Preda, Raffaele Falco, Chiara Tognola, Marco Carbonaro, Sara Vargiu, Michela Gallazzi, Matteo Baroni, Lorenzo Gigli, Marisa Varrenti, Giulia Colombo, Gabriele Zanotto, Cristina Giannattasio, Patrizio Mazzone, Fabrizio Guarracini

**Affiliations:** 1 Electrophysiology Unit, De Gasperis Cardio Center, Niguarda Hospital, 20162 Milan, Italymarisa.varrenti@ospedaleniguarda.it (M.V.);; 2 Clinical Cardiology Unit, De Gasperis Cardio Center, Niguarda Hospital, 20162 Milan, Italy; 3 Department of Cardiology, Ospedale Magalini di Villafranca, 37069 Villafranca di Verona, Italy; 4 School of Medicine and Surgery, University of Milano-Bicocca, 20126 Milan, Italy

**Keywords:** cardiac implantable electronic device, remote monitoring, atrial fibrillation, ventricular arrhythmias, heart failure

## Abstract

Over the past decade, remote monitoring (RM) has become an increasingly popular way to improve healthcare and health outcomes. Modern cardiac implantable electronic devices (CIEDs) are capable of recording an increasing amount of data related to CIED function, arrhythmias, physiological status and hemodynamic parameters, providing in-depth and updated information on patient cardiovascular function. The extensive use of RM for patients with CIED allows for early diagnosis and rapid assessment of relevant issues, both clinical and technical, as well as replacing outpatient follow-up improving overall management without compromise safety. This approach is recommended by current guidelines for all eligible patients affected by different chronic cardiac conditions including either brady- and tachy-arrhythmias and heart failure. Beyond to clinical advantages, RM has demonstrated cost-effectiveness and is associated with elevated levels of patient satisfaction. Future perspectives include improving security, interoperability and diagnostic power as well as to engage patients with digital health technology. This review aims to update existing data concerning clinical outcomes in patients managed with RM in the wide spectrum of cardiac arrhythmias and Hear Failure (HF), disclosing also about safety, effectiveness, patient satisfaction and cost-saving.

## 1. Introduction

Cardiac implantable electronic devices (CIEDs) are medical tools with diagnostic and therapeutic role, able to detect brady or tachy-arrhythmias, regulate cardiac rate and rhythm, improve heart function and manage fatal arrhythmias. These devices include implantable loop recorders (ILRs), pacemakers (PMs), implantable cardioverter-defibrillators (ICDs) and cardiac resynchronization therapy (CRT) systems. In recent years, there has been an exponential increase in the use of CIEDs corresponding to the rise in patients affected by clinical complexities [1]. The crescent indication to CIEDs has been related to the increase in aged population, increased trend toward overdiagnosis and overtreatment, technological advances in CIEDs functions and expanded indications for both invasive cardiac interventions and pacing [2]. CIEDs are continuously updated technologically by electromedical companies and are capable of storing an ever-increasing amount of data and diagnostic information related to the device’s function, occurrence of arrhythmias, detection of physiological parameters, and indicators of cardiovascular homeostasis. The implementation of remote monitoring (RM) systems has completely revolutionized the clinical management of CIEDs carriers, providing significant results in terms of patient outcomes, medication adherence, patient engagement and satisfaction, personalized care, remote data availability, self-management of health conditions and cost reduction [3]. In addition, RM paved the way to early diagnosis of cardiac arrhythmias such as atrial fibrillation (AF) as well as early identification of hemodynamic changes in patients affected by HF, providing useful data for a prompt medical management [4]. From the first HRS Expert Consensus Statement on RM published in 2015 [5], several randomized and observational studies and large registries confirmed as well as expanded the role of RM among the wide spectrum of cardiac diseases. Currently, RM is recommended as a first-line strategy for CIED follow-up, according to most recent guidelines by American and European scientific societies [6,7]. This review aims to update existing data concerning clinical outcomes in patients managed with RM in the wide spectrum of cardiac arrhythmias and HF, disclosing also about safety, effectiveness, patient satisfaction and cost-saving.

## 2. Logistics and Rationale of Remote Monitoring

In the majority of patients carrying CIEDs, outpatient check-ups are generally scheduled every 3–12 months, depending on the center’s organization, patient complexity, device type and disease progression. This puts a significant burden on health care system in terms of costs and organization. Automatic RM changed this paradigm, providing a mechanism for continuous surveillance of an ambulatory population with CIEDs and virtually immediate alert-based notification of changes in device (or patient) condition. This is performed via diverse vendor-specific systems, all of which are based on the availability of a patient monitor capable of interrogating the CIED and sending the data to a central server where the information is decrypted and made available to the clinical staff members responsible for the patient’s care. RM comprises scheduled intervals of full remote device interrogation as a substitute for in-office visits, unscheduled remote monitoring transmission for predefined alert events and patient-triggered interrogation after encountering a real or perceived clinical event. This novel approach to patient follow-up significantly improved the time to diagnosis and the subsequent time to therapy for various of clinical events [8], thereby reducing resource consumption and expenses both for patients and for healthcare systems. Traditional outpatient check-ups are replaced without compromising safety [9,10,11]. The introduction of RM into clinical practice has underscored the need for new organizational models to manage the activities of various professionals involved in the diagnostic-therapeutic process and to ensure a precise definition of roles and responsibilities, traceability of actions, continuity of care, minimal resource consumption, patient satisfaction and acceptance. Moreover, it emphasized integration with traditional hospital and outpatient diagnostic and treatment pathways [12,13].

## 3. Safety

Numerous studies have shown relevant improvements in patient safety when compared to traditional follow-up. A study on RM of ICDs carriers reported 41% clinical events, 3% had a technical event (regarding system integrity) over 10 months follow-up. Of note, 60% of events occurred at the first month after the last outpatient visit [14]. A retrospective analysis of more than 3 million transmissions from over 11,000 patients with PM or ICD found a time interval between the last follow-up and an event reported by RM of 26 days [15]. Considering a standard average interval of 6 months between outpatient follow-ups, it is evident that RM allows for a gain of 154 days per event (64 days per event in patients monitored every 3 months); a time interval comparable to the 144 days reported in the COMPAS study [16]. Similar findings were shown by the TRUST study [17], which demonstrated a reduction of load of in-clinic evaluations by almost 50% (principally by reducing non-actionable routine device evaluations) while maintaining patient safety, as well as a reduction in the median time to evaluate a clinically relevant event to 3 days vs. 30 days of traditional follow-up. The CONNECT study also showed a decrease in the time for a clinical decision post-event from 22 days in standard follow-up to 4.6 days with RM [18].

## 4. Mortality

**Figure 1:**
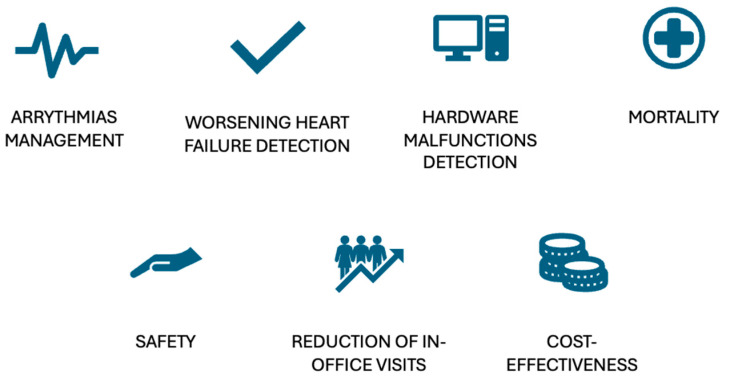
Benefits of Remote Monitoring.

Several studies aimed to evaluate all-cause mortality in patients followed with RM. In a 2014 study involving HF patients with high-voltage devices (ICD or CRT-D), those assigned to Home Monitoring™ (Biotronik, Berlin, Germany) exhibited reduced mortality compared to those receiving only quarterly in-office visits [19]. ECOST trial [11] reported a large reduction in the number of delivered shocks, charged shocks and the rate of inappropriate shocks in RM patients compared to controls. To be note, every ICD-shock negatively impacts on patient outcome [20]. Despite early promise from real-world data [21,22], randomized controlled trials (RCTs) and meta-analyses evaluating the effect of RM on survival have been largely neutral [23,24], although associated with a marked reduction in planned hospital visits and lower costs) [25]. Several RCTs found no mortality benefit compared to usual care [18,26,27,28,29]. RM provided a significant reduction in all-cause mortality only if performed by daily monitoring (OR = 0.65; 95% CI: 0.45–0.94; *p* = 0.021) [24]. Finally, other observational and cohort studies reported a survival benefit provided by RM, as demonstrated in the ALTITUDE survival study [13] and the survival analysis from the Merlin database [30]. Figure 1 represents all benefits provided by RM.

## 5. Heart Failure

The hospitalization rate for HF remains high despite novel therapeutic approaches and each acute event worsens life expectancy [31] as well as being associated with an 8-fold and 9-fold increase in mortality and hospital readmissions [32]. According to a 2010 study estimating overall HF patients eligible for ICD implantation up to 54% [33], this proportion is expected to be strongly increased during the last decade due to expanded indications for CRT including novel conduction system strategies [34] and the availability of CIED capable to entail the collection of several physiologic parameters using technologies specifically developed for the HF population (i.e., intra-thoracic impedance, heart rate and heart rate variability, physical activity, minute ventilation, heart sound amplitude in addition to arrhythmia detection, and statistics on the percentage of biventricular stimulation in CRT patients) [35]. Intrathoracic impedance monitoring is the current most used modality for the detection of impeding HF, in particular OptiVol fluid index predicted worsening cardiac events with a high detection rate, although false positive have been reported [35,36]. Telemonitoring was introduced with the hope of decreasing at home decompensation, stretching the capacity of HF clinic staff to manage growing caseloads and enabling patients to interpret and respond to their own physiological data, promoting self-empowerment and care [37]. REM-HF [38] is the largest randomized study evaluating the role of RM in preventing death from any causes and hospitalization for cardiovascular reasons in HF patients carrying a CIED. The trial enrolled 1650 patients randomized to active RM or usual care for a median of 2.8 years, without finding significant difference in primary outcome (HR 1.01; 95% confidence interval (CI) 0.87–1.18). A pre-specified analysis focusing on patients who had device-detected AF in the first year of follow-up showed a greater volume of clinical activity in patients with AF, in particular for the persistent type [39].

Despite RM did not result in reduction of the primary outcome HF patients with AF in that study, RM findings may result useful in medical management of these patients such as in correlating symptoms, in quantify AF burden and in early treatment with oral anticoagulation [39]. RM may consider various parameters to evaluate the course of the disease (i.e., average heart rate, atrial arrhythmias, exercise and daily activity, heart rate variability, transthoracic and intracardiac impedance, apnea scan, etc.), although no parameters has demonstrated sufficient diagnostic power to guide HF management individually. Algorithms capable of performing a multiparametric evaluation are currently available with the aim of increasing sensitivity and specificity, seeking to identify patients at higher risk of hospitalization for worsening HF. In this setting the MultiSENSE trial enrolled 900 patients with CRT followed for one year. The device software allowed simultaneous assessment of various physiological parameters that were combined into a multi-sensor alert algorithm known as HeartLogic. The HeartLogic algorithm, either alone or in addition to measuring the blood levels of NT-proBNP, was able to identify time-intervals when patients are at significantly increased risk of worsening HF [40]. Despite optimistic results provided by some studies, the real impact of RM on hard endpoints is currently uncertain, as demonstrated by a recent meta-analysis of 10,981 patients from 29 trials reporting significant but modest and variable benefit of this type of telemonitoring to reduce HFH and improve quality of life [41].

## 6. Diagnosis, Management and Follow Up of Cardiac Arrhythmias

### 6.1. Atrial Fibrillation

RM is largely employed in patients at risk of both ventricular or supraventricular arrhythmias, particularly for AF, that is the most common cardiac arrythmia, associated with a reduce quality of life and an increased long-term risk of stroke, HF and all-cause mortality [42]. In a non negligible proportion of patients, ischemic stroke or systemic embolism can be the first clinical event [43] and gender differences in disease development and embolic risk have been documented [44]. In those without a prior diagnosis, RM may play a fundamental role in risk stratification and early detection. The CRYSTAL-AF demonstrated the superiority of continuous monitoring by ILR compared to the conventional follow up with Holter ECG 24 h for AF occurrence at 12 months in patients with cryptogenic stroke (12.4% vs. 2.0%) [45]. Moreover, RM permits early detection of frequent premature atrial complexes (PACs), that well known independent predictors of sustained atrial tachycardia and AF. A study reported that daily number of PACs significantly increased in the 5 days preceding the AF occurrence and that the risk of AF was significantly higher in patients with a relative increase of the daily PACs over 30% compared to ten preceding days [46]. In particular, patients with or without AF diagnosis have a different arrhythmic atrial pattern with a statistically significant difference in daily number of PACs (1226 and 142 PACs per day), reflecting the different degree of individual atrial remodeling. High-frequency atrial events (AHRE) are defined as atrial tachyarrhythmia episodes registered by CIEDs with a frequency superior to 175 bpm and a duration over 5 min without clinical ECG-documented atrial fibrillation [42]. The presence of AHRE longer than 5 min was found to be an independent predictor of total mortality and non-fatal stroke [47,48]. AHRE are frequently encountered in patients undergoing PM/ICD implantation, up to 30% in AF-free patients [49,50]. Among these, 16% experience clinical atrial tachyarrhythmia identified on 12-lead ECG and 4% develop a stroke/systemic embolic event in the following 2.5 years [49]. Based on these findings, it was calculated that the presence of AHRE increases the patient’s risk of developing AF by 5 times and approximately 2.5 times the risk of experiencing a stroke/systemic embolism compared to patients without AHRE. A pooled analysis assessing more than 10,000 patients affected by paroxysmal or persistent AF reported the AF burden detected by CIED as an independent predictor of ischemic stroke, particularly in cases of AF burden > 1 h (HR 2.11; *p* = 0.008) [51]. Of note, also the threshold of ≥5 min was statistically significant (HR 1.76, *p* = 0.041). According to guidelines, anticoagulation is recommended in cases of AHRE > 24 h while there is controversy on the net benefit of anticoagulation for AHRE of lesser duration, suggesting a patient-tailored approach considering the individual stroke and bleeding risk [42,52]. Recently, two large randomized trials have conducted to evaluate the role of anticoagulant therapy in short AHRE (at least 6 min). ARTESIA trial [53] (4012 participants) showed that Apixaban reduced the risk of stroke or systemic embolism compared with aspirin, while the NOAH-AFNET 6 [54] (2536 participants) failed to show a protective effect of edoxaban (1.1%/year stroke rate in anticoagulation-free group vs. 0.9%year in the anticoagulation one). However, a recent meta-analysis of these two trials provides high-quality evidence that oral anticoagulation with Edoxaban and Apixaban reduces the risk of stroke in patients with any device-detected AF ([RR] 0.68, 95% confidence interval [CI] 0.50–0.92), thus reinforcing the role of RM in reducing morbidity [55]. RM provides a chronologic plot of all AF events and their individual durations, accompanying intra-cardiac electrograms, AF burden trends, and associated ventricular rates. This has the potential to improve the therapeutic management of patients treated with a rhythm control strategy, leading to real-time therapeutic changes. In patients with a CHADsVASC score < 2 and no more documentation of AF, OAC withdrawal may be discussed in order to minimize the hemorrhagic risk. This option was investigated in a large experience of 831 patients undergoing AF ablation [56], reporting a very low post-procedure stroke risk (0.06%/year). Although this therapeutic approach is currently off-label, association with a continuous rhythm monitoring may further enhance its safety [57]. Catheter ablation is superior to antiarrhythmic pharmacological treatment in maintaining sinus rhythm among patients with symptomatic, drug-refractory AF [58]. In this setting, a continuous heart rhythm monitoring provides a realistic measure of the therapeutic effectiveness of the ablation. In DISCERN-AF trial [59], a large multicentric prospective study, the ratio of asymptomatic to symptomatic AF episodes increase from 1.1 before to 3.7 after catheter ablation. These results underline one-more time the low diagnostic power of symptoms alone. The time of ablation to recurrence is a major indicator of long-term AF recurrence. Indeed, despite AF recurrence during the blanking period is not to be classified as a treatment failure, two large trials (STOP-AF [60] and STAR-AF [61]) respectively using cryoablation and radiofrequency for Pulmonary Vein Isolation (PVI), demonstrated that early recurrence of AF during the first three months was strongly associated to its late recurrence (LR). The prognostic power of the blanking period on LRs was further confirmed by another study of 477 patients undergoing AF ablation reporting a 4-fold higher risk of LR [62]. Intriguingly, some studies have shown that recurrence in early blanking period (within 1–2 months) was less predictive of LR compared to those occurring in the third month [63]. Finally, since the rhythm control strategy is the current treatment associated with the best outcome, RM of sinus rhythm is also of utmost importance in long-term follow up [64].

### 6.2. Ventricular Arrhythmias

**Figure 2:**
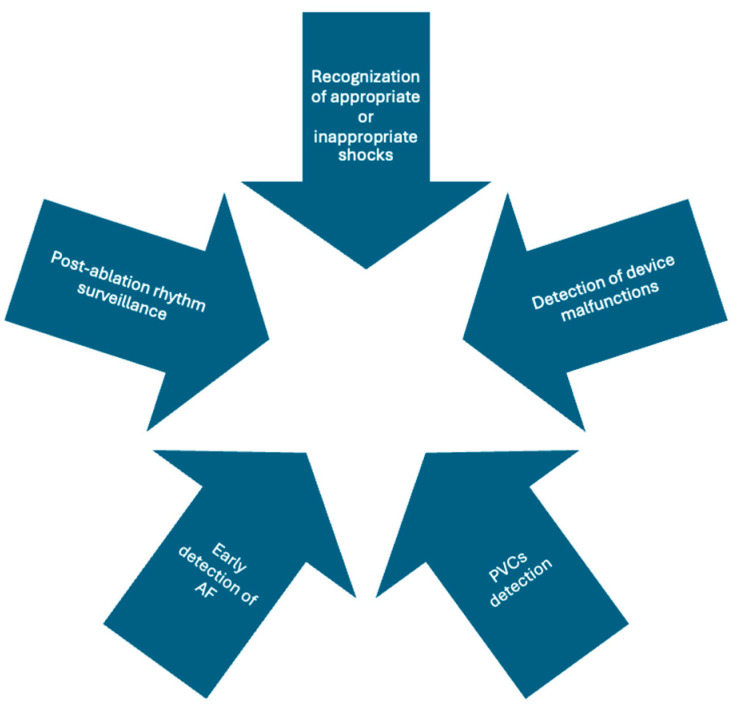
Benefits of RM in terms of Arrhythmias Management.

Premature ventricular contractions (PVCs) have been associated with mortality and HF regardless the presence of structural heart disease [65]. Frequent PVCs may be a first marker of underline abnormal cardiac substrate but can also be responsible of left ventricular function impairment, particularly if the burden overcome 15–20% of total beats [66]. Holter ECG 24 h is the usual ambulatorial strategy to define PVC burden and morphology, with the non-negligible limit of the restricted monitored time. On the other hand, CIED provide a long-lasting monitoring by an automated PVC count algorithm that may overestimate PVC burden in case of coexistent AF or intermittent atrial undersensing. In a monocentric study, when CIEDs (PM and ICD) were compared to an ambulatorial monitoring they showed a poor sensitivity (0.16) but a high specificity (0.99) in detecting PVCs [67]. A sub analysis of the MADIT-CRT (Multicenter Automatic Defibrillator Implantation Trial with Cardiac Resynchronization Therapy) demonstrated the link between the overall burden of premature contractions (both atrial and ventricular) and subsequent low percentage of biventricular pacing with lower rate of reverse remodeling, worsening of HF, death and ventricular tachyarrhythmias [68]. A sub-analysis of the UMBRELLA multicentric registry that included 1268 carriers of CRT showed a 11% prevalence of high PVC burden (>200/h) transmitted by RM [69]. Since a high percentage (>98%) of biventricular (BiV) pacing is necessary in order to derive maximum benefit from CRT [70], aggressive treatment of PAC and PVC is deemed necessary by pharmacotherapy or catheter ablation. In these patients, RM is crucial to identify both potential non-responders to CRT due to the higher number of PACs/PVCs and the response to therapy without the need for in-hospital visits. Furthermore, in the case of ventricular arrhythmias with or without intervention by the ICD, RM allows for a prompt assessment of the appropriateness and effectiveness of therapy. The TRUST study has demonstrated that RM allows for earlier analysis of ventricular fibrillation events (1 day vs. 36 days) and ventricular tachycardia events (1 day vs. 28 days) [17]. RM can also prevent inappropriate shocks for supraventricular arrhythmias because timely recognition of inappropriate classification of arrhythmias can guide prompt device reprogramming or other therapeutic interventions. Avoiding unnecessary (including abortive) capacitor charges allows for battery savings. The ECOST study [11] demonstrated a reduction in the number of delivered shocks by 72%, capacitor charges by 76%, and inappropriate shocks by 52%. In conclusion, ILR implantation aimed at continuous rhythm control monitoring may represent a reasonable alternative to ICD in patients with arrhythmogenic cardiomyopathy, particularly in evolving cases or those with indetermined/lower risk of sudden cardiac death [71]. This is especially relevant for individuals with left/biventricular dysplasia, for which current guidelines are still uncertain [72]. Table 1 reports major findings of currently published trials on RM. Figure 2 represents the benefits of RM in terms of arrhythmias management.

## 7. Healthcare Optimization and Cost Saving

CIEDs are composed by sophisticated electronic and mechanical components with an inherent risk of device-related complications. Any compromise in system integrity demands prompt evaluation. The recommendation of Heart Rhythm Society is to include patients with a CIED component under advisory or recall in RM [6]. Indeed, RM allows for a timely response to the detection of a CIED malfunction and quick recall, permitting a reasonably conservative approach and reserving replacement for selected cases [75]. Given the unpredictability of lead fractures, daily monitoring can detect problems early, minimizing the risks of adverse clinical events. In a study on ICD carriers that encountered a malfunction on follow up, the composite outcome of inappropriate shocks and symptomatic inhibition of stimulation was significantly lower in the group of patients followed with RM (27.3% vs. 53.4%, respectively; *p* = 0.04) [76]. Despite sophisticated algorithms to detect malfunctions or arrhythmias, a non-negligible proportion of false positives and redundant transmissions, up to 40%, were reported [77,78]. Indeed, RM of CIEDs generates a large volume of transmissions, which contributes to data deluge and therefore needs effective and efficient triage to identify alerts in which active clinical intervention is needed. Alert burden and data deluge from CIEDs are a challenge in the practice of electrophysiology, underlining the crucial role of expert revaluation of any automated alert and the need for alert filtering algorithms improvement. Nevertheless, RM demonstrated to significantly reduced unplanned visits, emergency room admissions, and hospitalizations without an increase in adverse events [79]. Half of the outpatient visits could be avoided without compromising patient safety according to a study [80]. Another study reported that only 6% of planned outpatient visits may result in device reprogramming or patient hospitalization, consequently, 94% of checks would be could be replaceable by RM [10]. In the TRUST study, the average number of hospital visits was 2.1 in the RM group compared to 3.8 patient/year (*p* < 0.001) in patients with exclusive in-office follow-up. At 12 months, outpatient visits were reduced by 45% and 86% of all follow-ups were conducted exclusively using RM without differences in adverse events and mortality [17]. The Insync ICD Italian Registry highlighted a marked reduction in the number of interrogations with subsequent device reprogramming in the first 6 months of follow-up, as the device was optimized immediately after implantation, and programming was not subsequently modified [81]. This issue was also extended to PM recipients in a randomized study involving 538 patients followed for 18 months that reported early diagnosis of adverse events (median 144 days earlier than conventional follow-ups) in the RM group with drastic reduction in the number of outpatient visits [16]. RM is also associated with high acceptance and satisfaction compared to out-patients visits [79], without crossover to conventional follow-up. In those studies, only a minority (<5%) raised concerns about privacy, fear of losing human contact physicians and doubts about technology [17]. However, to overcome these concerns, a comprehensive explanation of the expected benefits and detailed information about the organizational model are sufficient [82]. The recent European REMOTE-CIED study is the first randomized trial primarily designed to evaluate the effect of RM on patient-reported outcomes in the first 2 years after implantation of an ICD. No differences in patient-reported health status and ICD acceptance between the RM group and out-patient group were reported [74]. The cost-effectiveness of RM has been demonstrated by numerous randomized and non-randomized studies. The Connect study demonstrated, in a cohort of approximately 2000 patients with bicameral and biventricular defibrillators, a reduction in time to clinical decision and a shorter average hospitalization time for cardiovascular events, resulting in an estimated saving of around $1659 per hospitalization in the American healthcare system [18]. The prospective, multicenter study EVOLVO assessed the clinical-economic impact of RM on HF carriers of ICD or CRT-D demonstrating a 36% reduction in urgent visits and a 23% reduction in all hospital admissions for cardiac or device-related causes, leading to a 25% reduction in in the annual cost for accessing care [28]. Similar results were obtained in the multicentric MoreCare study that showed a 38% reduction in the burden of resources used for cardiovascular reasons in the RM arm, resulting in approximately €12,000 in savings for the healthcare system for every 100 patients managed over a span of 2 years [83].

## 8. Future Prospective

In recent years, several studies evaluated the growing diagnostic role of artificial intelligence (AI) in cardiovascular disease, in particular algorithms for the detection, prediction, and management of atrial fibrillation (AF). Actually, AI can detect AF with a high accuracy using 12-lead electrocardiograms with false-positive deriving form detection of PACs and from respiratory arrhythmia [84]. When using a single-lead electrocardiograms the performance is moderately reduced [85], suggesting that this model may be employed also for ILRs and wearables. Although the application of AI to cardiac electrophysiology is still nascent, a definite diagnosis of AF currently needs to be confirmed by a physician, but AI may be helpful as initial filter to reduce the number of false-positives, above all for RM when using ILRs [86]. Ongoing studies are evaluating new algorithms for detecting with better accuracy AF through 12 or single-lead ECG.

## 9. Conclusions and Limits

In light of the robust data from scientific literature and continuous technological advances, RM demonstrated to vastly replace out-patient follow-up without impacting safety and effectiveness of the CIED. This resulted also in reduction in both medical and non-medical times for handling clinical complexities, with a significantly favorable impact on the patient’s disease follow-up. Furthermore, RM provided additional benefits in diagnosis of occulted arrhythmias as well as therapeutic efficacy of medical/interventional management. Despite it all, although optimistic results provided by several studies, there is no a well-defined role of RM on the hard endpoints of HF such as reduction of mortality and hospitalization. This could be an important future direction for RM.

Current expert consensus recommends RM as a part of the standard care of patients with CIEDs.

## Figures and Tables

**Table 1:** Evidence from randomized clinical trial regarding benefits of RM versus standard of care.

Study	Cohort, CIED Carried, Mean Follow up	Major Findings
TRUST (2010) [17]	1339, PM + ICD, 15 months	Reduction in median time to evaluate clinically relevant event (3 days vs. 30 days, *p* < 0.01)
COMPAS (2011) [16]	538, PM 18 months	Reduction in MAE (−1.8%, *p* < 0.01 for non-inferiority) and interim ambulatory visits (−56%, *p* < 0.01)
CONNECT (2011) [18]	1997, ICD, 15 months	Reduction in median time to evaluate clinically relevant event (4.6 days vs. 22 days)
EVATEL (2011) [73]	1501, ICD, 12 months	Non-inferiority goal for death, cardiovascular hospitalization and ineffective or inappropriate therapy not reached
ECOST (2012) [11]	433, ICD, 24 months	Reduction in MAE (−3%, *p* < 0.05 for non-inferiority)
EVOLVO (2012) [28]	200, ICD, 16 months	Reduction in emergency department/urgent in-office visits and total healthcare use (−35%, *p* = 0.005)
MORE-CARE (2013) [27]	154, CRT-D, 12 months	No difference in death, cardiovascular, and device-related hospitalization
IN-TIME (2014) [19]	716, ICD, 12 months	Reduction in HF worsening (−18.9%, *p* < 0.05)
OptiLink (2016) [29]	1002, ICD/CRT, 19 months	No difference in all-cause death and cardiovascular hospitalization
REM-HF (2017) [39]	1650, ICD, 33.5 months	No differences in death from any cause or unplanned hospitalization for cardiovascular reasons
MultiSENSE (2017) [40]	900, CRT-D, 12 months	HeartLogic multisensor index and alert algorithm showed 70% Sensitivity to detect HF events and 1.47% unexplained alerts per patient-year
REMOTE CIED (2019) [74]	595, ICD, 24 months	No differences in incidence of mortality and hospitalization

Abbreviations: CRT-D, cardiac resynchronization therapy-defibrillator; ICD, implantable cardioverter defibrillator; MAE, major adverse events; PM, pacemaker; RM, remote monitoring.

## Data Availability

Not applicable.
